# PPM-YOLOv11: Improved YOLOv11n-Based Algorithm for Small-Object Detection in Aerial Images

**DOI:** 10.3390/s26072030

**Published:** 2026-03-24

**Authors:** Yuheng Yang, Haiying Zhang, Xiaoya Wang

**Affiliations:** China Electronics Technology Group Corporation 54th Research Institute, Shijiazhuang 050081, China; yangyuheng180@163.com (Y.Y.); xy-wang@bupt.edu.cn (X.W.)

**Keywords:** occlusion awareness, YOLOv11, small-object detection, UAV, feature fusion

## Abstract

To address the challenges in drone aerial image target detection—including the loss of critical information on small objects during multiple subsampling operations, the disappearance of minute target features, and insufficient detection accuracy due to dense occlusion interference—we propose PPM-YOLOv11, an improved target detection algorithm based on YOLOv11n. The C3K2_PPA module integrates parallelized patch-aware attention with the C3K2 backbone network to better preserve critical information on small objects. A multi-scale detection head P2 specifically designed for detecting ultra-small objects ranging from 4 × 4 to 8 × 8 pixels is introduced. A high-resolution feature layer is added to the neck network to enhance detection accuracy with respect to ultra-small objects from a drone’s perspective. Adding the MultiSEAM module to the neck network enhances detection of occluded small objects by amplifying feature responses in unobstructed regions and compensating for information loss in occluded areas. Experiments on VisDrone2019 and SIMD datasets demonstrate our algorithm achieves a 40.9% mAP50 on VisDrone2019, surpassing the baseline YOLOv11n by 9.3 percentage points. On the SIMD dataset, the mAP50 reached 82.0%, surpassing the baseline network by 3.9 percentage points.

## 1. Introduction

With the rapid advancement and widespread adoption of drone technology, drones’ flexible maneuverability, low operational costs, and unique aerial perspective have enabled extensive applications across public safety surveillance, urban-planning management, agricultural crop monitoring, infrastructure inspection, natural disaster assessment, and film/entertainment production [1]. UAVs have become an indispensable component within integrated air–ground–space information-sensing networks. Across these applications, enabling UAVs to “interpret” the vast array of visual data they capture—achieving autonomous environmental perception and understanding—has emerged as a core technological challenge [2]. Aerial target detection for UAVs stands as a focal point in the convergence of computer vision and UAV technology [3]. The core task involves precisely locating and identifying specific targets (pedestrians, vehicles, buildings, etc.) within aerial images or video sequences, providing data support for higher-level decision-making and control.

However, unlike in conventional ground-level or close-range image detection tasks, UAVs typically operate at high altitudes, employing a unique operational mode that poses a series of formidable challenges regarding their object detection technology [4]. First, an extremely small target scale and dense distributions are the most prominent characteristics. Due to the long shooting distance, targets in images—such as pedestrians on roads or cars in parking lots—occupy a very limited pixel area [5]. In the authoritative public benchmark VisDrone2019, the dimensions of the annotated bounding boxes for a large number of targets are concentrated within the ultra-small scale range of 4 × 4 to 8 × 8 pixels [6]. These minute objects contain sparse visual features and blurred textural details, making them easily obscured within complex backgrounds. Second, perspective shifts are extreme [7]. During flight, UAVs undergo continuous dynamic changes in altitude, angle, and orientation, resulting in significant scale and shape variations for objects of the same category across images [8]. Furthermore, scene complexity is high. Aerial image backgrounds encompass diverse terrains, including urban blocks, farmland, highways, and rivers. Targets frequently suffer severe occlusion due to building obstructions, tree cover, or mutual overlap [9]. This not only compromises feature integrity but also substantially increases the difficulty of target separation and recognition [10].

Thus far, deep-learning-based object detection algorithms, particularly models like YOLO (You Only Look Once) [11], Faster R-CNN [12], and DETR [13], have achieved remarkable success on general-purpose detection datasets such as COCO [14], ImageNet Detection [15], and PASCAL VOC [16]. Among these algorithms, the YOLO series models have gained particular favor in both industry and academia due to their excellent balance between speed and accuracy [17]. YOLOv11 continues this design philosophy, achieving new heights in efficiency and performance. However, when these general-purpose detection models—which excel on natural images—are directly transferred to the specific domain of drone aerial photography, their inherent network architectures often prove inadequate, resulting in significant performance degradation [18]. Fundamentally, the design of general-purpose detection models does not sufficiently account for core challenges in drone aerial scenarios, such as ultra-small objects, extreme scale variations, and dense occlusions [19].

Specifically, existing models exhibit three primary shortcomings:

1. Information decay in deep networks [20]: To extract high-level semantic features and expand the receptive field, modern convolutional neural networks typically employ strided convolutions or multiple downsampling pooling layers (e.g., 32× or even 64× dimensional reduction). While effective for large-scale targets, this process dilutes and nearly extinguishes the limited feature information regarding ultra-small objects (often occupying only a dozen pixels) through successive downsampling. Consequently, effective target signals cannot be preserved in deep feature maps, constituting a fundamental cause of small-target detection failures [21].
2. Insufficient detection head sensitivity for minute objects [22]: Standard detection heads (e.g., YOLO’s Head) typically operate on low-resolution feature maps resulting from multiple downsampling stages. While these feature maps contain rich semantic information, they suffer from severe loss of spatial detail, making precise localization and classification of minute objects challenging [23]. Models lack specialized mechanisms for detecting extremely small-scale objects.
3. Limited modeling capability for occlusion issues: In dense scenes, mutual occlusion between objects generates numerous incomplete or partially visible instances [24]. General models typically rely on holistic appearance features during training. When occluded, these features weaken and become easily confused with backgrounds or other objects [25]. Existing methods lack effective mechanisms with which to explicitly compensate for occlusion-induced information loss and enhance the value of remaining visible parts.

In summary, developing a drone aerial target detection algorithm that effectively addresses information decay, extremely small and dense scales, and severe occlusion not only holds significant theoretical value—advancing foundational computer vision research on small-object detection and occlusion handling—but also addresses urgent practical demands. It represents a critical technological bottleneck for enhancing drone autonomous intelligence and unlocking broader application scenarios.

To address this, this paper proposes PPM-YOLOv11, an improved drone aerial target detection algorithm based on YOLOv11n. The main contributions of this work are threefold:

1. Integration of the Parallel Patch-aware Module (PPM) [26] with the C3k2 module of the backbone network to form the C3k2_PPA module—This module is positioned on the critical path of the network’s forward propagation, enabling the early preservation of key information for small objects during feature extraction.
2. Introduction of a multi-scale P2 detection head [27] specifically tailored for detecting ultra-small objects ranging from 4 × 4 to 8 × 8 pixels—By integrating high-resolution feature layers into the neck network and employing a parallel branch architecture to optimize bounding-box regression and classification tasks independently, the model achieves significantly enhanced detection accuracy for extremely small objects.
3. The addition of the Multi-scale Squeeze and Excitation Attention Module (MultiSEAM) [28] to the neck network—This module adaptively enhances feature responses in non-occluded regions while compensating for information loss in occluded areas, comprehensively boosting detection capabilities in dense scenes.

Experimental results obtained using the VisDrone2019 and SIMD datasets demonstrate that the proposed PPM-YOLOv11n algorithm achieves significant detection accuracy improvements over baseline models, effectively validating its effectiveness and advanced capabilities for small-object detection in drone aerial photography tasks.

## 2. Materials and Methods

### 2.1. Baseline Model

As a representative model of the YOLO (You Only Look Once) series [29], YOLOv11 inherits the core philosophy of treating object detection as a single regression problem. Through its end-to-end network architecture, it achieves direct prediction from image pixels to object bounding boxes and categories, striking an exceptional balance between speed and accuracy. Very recently, YOLO-RECAP [30] was proposed to enhance feature representation through reassembly with channel attention, demonstrating improved performance on general object detection tasks. This method shares the goal of feature enhancement with our C3K2_PPA module but differs in its approach: YOLO-RECAP focuses on channel-wise attention for feature reassembly, whereas our work emphasizes parallel patch-aware attention for proactive preservation of fine-grained details in ultra-small objects. Both methods highlight the ongoing interest in improving YOLO-based architectures, but our approach is specifically tailored to the unique challenges of UAV-based small-object detection, including extreme scales and dense occlusions. The comparative results on VisDrone2019 and SIMD datasets further validate the effectiveness of our design choices for aerial imagery scenarios.

To accommodate varying demands for efficiency and performance across different application scenarios, YOLOv11 offers five specifications, ranging from lightweight to high-precision: YOLOv11n, YOLOv11s, YOLOv11m, YOLOv11l, and YOLOv11x. These models share a unified base architecture, primarily scaled by adjusting the network’s depth and width.

**Figure 1:**
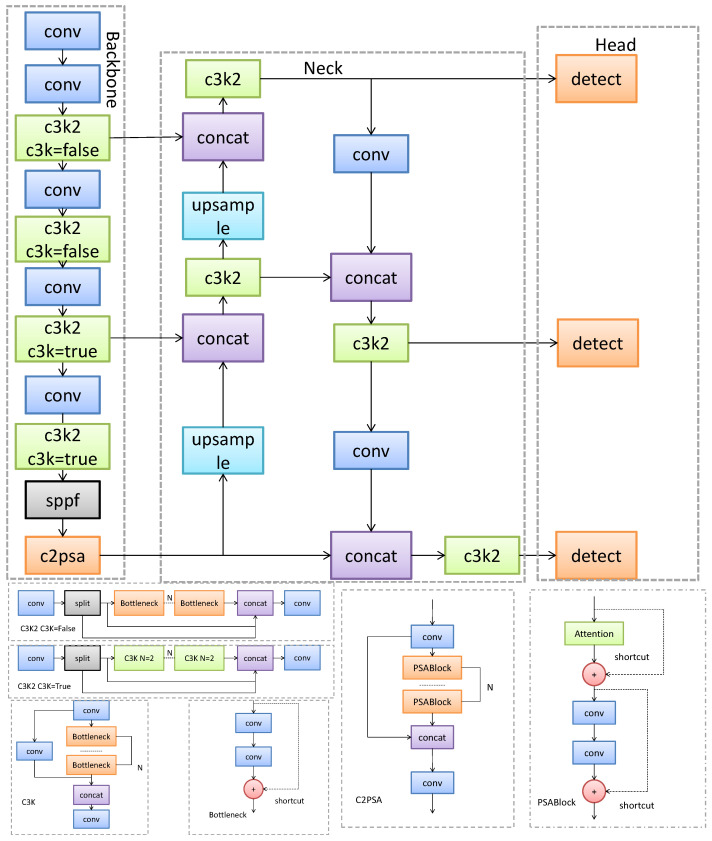
YOLOv11 network architecture.

As shown in Figure 1, YOLOv11’s architecture comprises three main components: the backbone network, neck network, and detection head. The backbone network handles multi-level feature extraction, featuring key modules including Conv modules for basic feature transformation; an enhanced C3K2 module introducing variable kernel sizes and channel separation strategies to boost feature expressiveness; and SPPF modules efficiently aggregating multi-scale context through serial pooling. The neck network, based on an optimized feature pyramid structure, integrates features from different depths of the backbone network. This effectively combines shallow-layer detail information with deep-layer semantic information, enhancing the model’s ability to detect objects at multiple scales. The detection head employs an anchor-free design, eliminating predefined anchor boxes. It directly predicts the center point offset and width/height of objects, simplifying the detection process and reducing hyperparameter dependency.

Balancing real-time performance and accuracy requirements, this study selects the lightest YOLOv11n version as the baseline model for subsequent research and improvements.

### 2.2. PPM-YOLO

**Figure 2:**
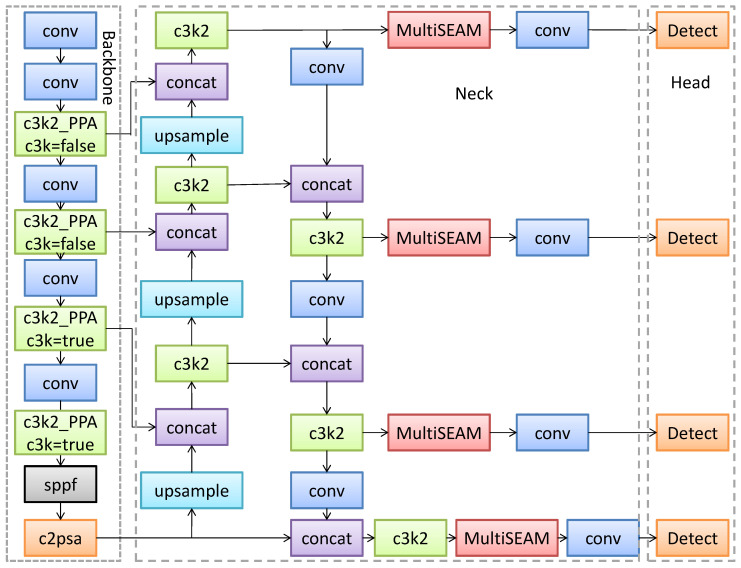
PPM-YOLO network architecture.

To address the formidable challenge of detecting ultra-small objects in drone aerial imagery, this paper proposes an enhanced model based on the YOLOv11n architecture—PPM-YOLO. Rather than a simple stacking of modules from the baseline model, this approach constructs a systematic solution featuring interlinked, synergistically enhanced components. By introducing targeted innovative modules at different critical stages of the network, PPM-YOLO achieves end-to-end optimization from feature extraction and fusion to final prediction. Its core design philosophy centers on “proactive feature retention, adaptive enhancement, and specialized detection.” The overall model architecture is illustrated in Figure 2, and its workflow and structural advantages can be systematically explained as follows.

PPM-YOLO inherits the efficient single-stage detection framework and Backbone-Neck-Head architecture from the YOLO series, but incorporates specialized designs tailored for small-object drone scenarios in each component. The model takes standard RGB images as input. After preprocessing steps like Mosaic data augmentation and adaptive image scaling, images are uniformly resized to the network’s input dimensions. They first enter the enhanced backbone network. Unlike the baseline model, this version replaces multiple standard C3k2 modules at key positions with a custom-designed C3k2_PPA module (core: Parallel Patch-Aware module). This module acts as the network’s “front-end optimizer,” proactively and efficiently capturing and preserving the fine texture and spatial information of small objects during the early and mid-stages of forward propagation. This approach curtails information decay caused by multiple downsampling operations at its source, providing higher-quality, more detailed multi-scale feature maps for subsequent processing. The neck network employs an extended feature pyramid network for multi-scale feature fusion. Its uniqueness lies in two aspects: First, it extends the fusion path upward, integrating higher-resolution feature layers from the backbone network to generate a P2 output layer specifically designed for small objects. Second, it introduces a multi-scale squeezed incentive attention module in parallel before feeding all layers from P2 to P5 into the detection head. This module acts as a “feature calibrator,” adaptively recalibrating feature channel weights by fusing multi-scale contextual information. This enhances feature responses in non-occluded regions while compensating for information loss in occluded areas. The detector head employs an anchor-free paradigm, outputting predictions across four scales: P2, P3, P4, and P5. The newly added P2 detector serves as a “specialized P2 detection head.” Leveraging its high-resolution capability, it directly handles detection of ultra-small objects ranging from 4 × 4 to 8 × 8 pixels. Working in tandem with the other three detectors, it achieves precise coverage of all-scale targets from a drone’s perspective.

The three modules are designed to address distinct yet complementary challenges. The C3K2_PPA module, integrated into the backbone, proactively preserves fine-grained features from the early stages of the network, mitigating the information decay caused by successive downsampling. This ensures that high-quality representations of ultra-small objects are retained for subsequent processing. The P2 detection head, added to the neck, provides a dedicated high-resolution pathway specifically targeting objects in the 4 × 4 to 8 × 8 pixel range. It leverages the preserved fine details from the backbone and combines them with semantic information from deeper layers, enabling precise localization of extremely small targets. The MultiSEAM module, inserted before each detection head, adaptively enhances feature responses in non-occluded regions and compensates for information loss in occluded areas by fusing multi-scale contextual information. This is particularly important for dense scenes where occlusions are common.

Crucially, these modules work synergistically: PPA ensures that the P2 head receives high-quality shallow features, while MultiSEAM benefits from both the rich representations preserved by PPA and the multi-scale features from the neck. Conversely, without PPA’s foundational feature preservation, the P2 head and MultiSEAM cannot fully exploit their potential, as evidenced by the ablation study (Section 3.4). This holistic design addresses the core challenges of UAV-based small-object detection—information loss, extreme scales, and dense occlusions—in a unified manner.

### 2.3. C3K2_PPA Feature Fusion Module

**Figure 3:**
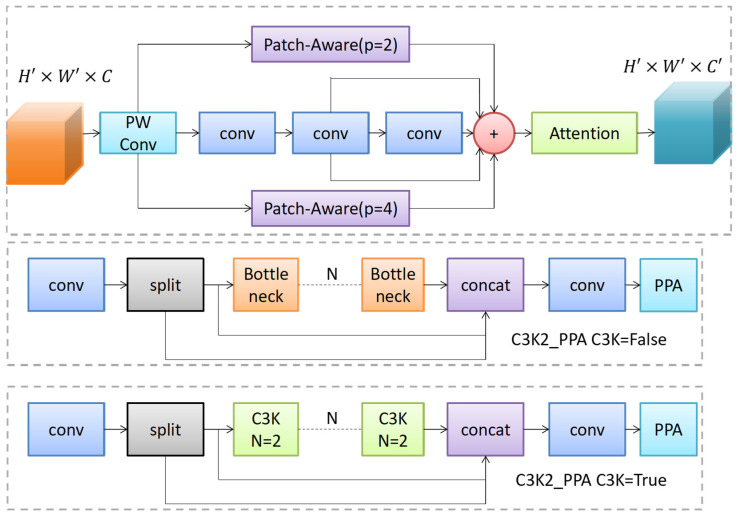
PPA Structure and C3K2_PPA Structure.

The Parallelized Patch-aware Attention Module (PPA) is an efficient feature enhancement architecture designed to capture multi-scale contextual information through a multi-branch design and attention mechanism, thereby enhancing the model’s perception of local details and global semantics. The core of the PPA module comprises two stages: multi-branch feature extraction and attention enhancement. Its structure, along with the C3K2_PPA architecture, is illustrated in Figure 3.

Given an input feature map $F_{in}\in R^{H\times W\times C}$, where H, W, and C denote the height, width, and number of channels of the feature map, respectively, the PPA module first performs a 1 × 1 convolution to adjust the channel dimension, yielding an intermediate feature $F^{\prime }\in R^{H\times W\times C^{\prime }}$. Subsequently, $F^{\prime }$ is fed into three parallel branches: The local patch branch divides the feature map into non-overlapping local patches using a small patch size (e.g., *p* = 2) and computes intra-patch attention weights to focus on subtle local features; The global patch branch aggregates contextual information over a larger spatial range by setting a larger patch size (e.g., *p* = 4), establishing associations between the target and its environment; the serial convolution branch consists of multiple concatenated 3 × 3 convolution layers, serving as the foundational feature extraction path to preserve the model’s basic representational capability. The outputs from the three branches are fused through element-wise summation:

(1) $$\tilde{F}=F_{local}+F_{global}+F_{conv}$$

where $F_{local}$, $F_{global}$, and $F_{conv}$ denote outputs from the local, global, and serial convolution branches, respectively. The fused feature $\tilde{F}$ is subsequently enhanced through channel attention and spatial attention mechanisms. Channel attention generates channel weights via the Efficient Channel Attention (ECA) module to amplify key channels; spatial attention produces a spatial weight map to highlight significant regions. Finally, the enhanced feature $F_{out}$ is output. Through this design, the PPA module effectively extracts and preserves multi-scale features, enhancing the model’s detail perception capability.

In drone aerial target detection tasks, objects (such as pedestrians and vehicles) in the VisDrone2019 dataset typically occupy only 4 × 4 to 8 × 8 pixels, qualifying as small objects. These targets face the following challenges in deep learning models:

Information loss: Due to multiple downsampling operations, spatial details and semantic information of small objects become severely diluted in deep layers, leading to incomplete feature representations and missed detections. Poor scale adaptability: Variations in UAV flight altitudes cause significant object scale differences, making it difficult for traditional convolutions to simultaneously capture details and context of extremely small objects. Computational efficiency bottlenecks: Directly introducing complex modules (e.g., PPA) may increase computational overhead, compromising real-time performance on UAV platforms.

These issues stem from the failure to proactively preserve critical information about small objects during feature extraction, preventing subsequent detection heads from effectively utilizing high-quality features.

To address the aforementioned challenges, this paper innovatively integrates the PPA module with the C3k2 module from the YOLOv11n backbone network, constructing the C3k2_PPA module. As a core component of PPM-YOLO, this module directly provides the following solutions to the key difficulties in this experiment:

The C3k2_PPA module integrates PPA’s multi-branch design at the source of feature extraction (i.e., early in the backbone network). By actively capturing details and contextual information of minute objects through local and global patch branches, it effectively mitigates information decay during subsampling. Compared to passive augmentation, this integration ensures small object features are continuously preserved during network forward propagation.

Through multi-branch feature extraction, the C3k2_PPA module simultaneously processes features at different scales. The local branch focuses on details while the global branch provides contextual information, enabling the model to adapt to the varying scales of targets in drone aerial photography.

The C3k2_PPA module integrates PPA as an internal branch within C3k2, enhancing only selected features. Although the PPA module increases computational cost (from 6.3 to 22.2 GFLOPs) by introducing multi-branch attention, the resulting accuracy gain (+4.5% mAP50) justifies this trade-off for precision-critical UAV applications. Moreover, the complete PPM-YOLO model (29.2 GFLOPs) remains more efficient than many existing detectors with comparable accuracy (e.g., PP-YOLOe-s at 15.5 GFLOPs but lower accuracy, or RS-DETR at 53.3 GFLOPs).

Experiments on the VisDrone2019 dataset demonstrate that the PPM-YOLO model incorporating the C3k2_PPA module significantly improves detection accuracy. Specifically, mAP@50 reach 36.1%, representing a 4.5 percentage point increase over the baseline YOLOv11n; mAP@95 reach 21.8%, showing a 3.5 percentage point improvement. Additionally, precision and recall improved to 48.5% and 35.3%, respectively. These results validate the C3k2_PPA module’s effectiveness in addressing small object information loss during drone aerial photography, providing richer feature representations that enhance overall detection performance.

Comparison with existing feature fusion methods: It is important to distinguish our C3K2_PPA from widely used feature fusion structures like PANet and BiFPN. While PANet and BiFPN focus on aggregating multi-scale features from different layers through top-down pathways, they do not change how features are extracted within each layer. In contrast, our C3K2_PPA enhances the feature extraction process itself by injecting attention directly into the C3k2 bottleneck. This allows the network to preserve details of ultra-small objects from the very beginning, before downsampling occurs. This “proactive preservation” strategy complements the inter-layer fusion performed later in the neck network.

### 2.4. P2 Test Head

In drone aerial imagery, numerous minute target instances—such as pedestrians and vehicles spanning 4 × 4 to 8 × 8 pixels—are present. Constrained by the visual blurring effect inherent in aerial overhead perspectives, traditional detection networks struggle to effectively capture the feature information of such minuscule targets. The original YOLOv11n model primarily detects heads at three scales: P3 (8× downsampling), P4 (32× downsampling), and P5 (64× downsampling). However, after multiple convolutions and downsampling operations, the limited pixel information of small objects becomes severely diluted. P4 (16× downsampling), and P5 (32× downsampling). However, after multiple convolutions and downsampling operations, the limited pixel information of small objects becomes severely diluted. Their feature responses gradually decay within the deep network layers, sometimes becoming completely obscured by complex backgrounds. This leads to degraded detection performance, manifested as high miss rates and high false positive rates.

**Figure 4:**
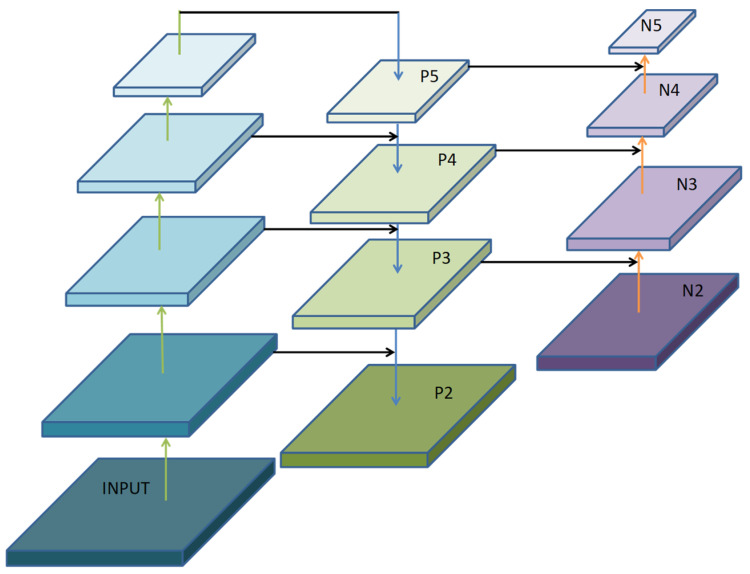
Four-Dimensional Feature Fusion Detection Network.

To address these issues, this paper introduces a dedicated high-resolution detection layer—P2—for small objects, building upon the existing P3, P4, and P5 detection layers. This creates a four-scale feature fusion detection network, whose architecture is shown in Figure 4. The core idea of this design is to leverage the rich spatial detail information retained in shallow network layers to provide more precise positional clues for detecting small objects.

The construction process of the P2 detection layer is as follows: Within the neck network, the P3 feature layer generated by the backbone network (downsampled by a factor of 8) undergoes upsampling to elevate its spatial resolution to one-quarter of the original input scale (i.e., P2/4). Subsequently, this upsampled feature undergoes cross-layer connection and feature fusion with corresponding feature maps from shallower layers of the backbone network (sharing identical spatial resolution). This design ensures the P2 detection layer incorporates rich semantic information from deep layers while preserving fine spatial details from shallow layers.

The introduction of the P2 detection layer enhances spatial detail. The P2 feature map possesses higher spatial resolution (typically 160 × 160 or larger), enabling more precise positioning information for edges, corners, and other features of ultra-small objects occupying only a few pixels—critical for accurate target localization. Through the collaborative operation of the four detection layers (P2, P3, P4, P5), the model achieves specialized detection for targets at different scales. Specifically, the P2 layer detects ultra-small objects ranging from 4 × 4 to 16 × 16 pixels, the P3 layer handles small-to-medium targets, while the P4 and P5 layers detect medium and large targets respectively, forming comprehensive scale coverage. This four-scale detection architecture enhances overall detection efficiency for targets with wide size distributions in drone aerial scenes through fine-grained scale division, without significantly increasing computational overhead.

Experimental results demonstrate that introducing the P2 detection layer significantly improves the PPM-YOLO model’s detection accuracy for ultra-small-scale targets on the VisDrone2019 dataset: mAP@50 reach 34.8%, representing a 3.2 percentage point increase over the baseline YOLOv11n; mAP@95 reach 20.5%, showing a 2.2 percentage point improvement. Additionally, precision and recall improved to 45.6% and 34.6%, respectively. This effectively addresses missed detections caused by insufficient feature information while maintaining robust detection performance for medium-to-large-scale targets.

Comparison with generic multi-scale heads: Adding a high-resolution P2 layer has been explored in some previous detectors. However, our implementation is specifically tailored to the extreme scale range (4 × 4 to 8 × 8 pixels) found in UAV imagery. Unlike generic multi-scale heads that simply attach a P2 layer to the feature pyramid, we carefully construct our P2 head by fusing upsampled P3 features (which carry semantic information) with corresponding high-resolution feature maps from the backbone (which retain spatial details). This ensures that the P2 head receives both the contextual and detailed information necessary for detecting ultra-small objects.

### 2.5. MultiSEAM Module

In drone aerial photography scenarios, target occlusion is one of the key factors affecting detection performance. Due to the presence of obstructions such as buildings and trees in urban environments, coupled with mutual overlapping between targets, certain target features become incomplete or entirely invisible. Traditional detection models struggle to effectively handle such occlusion scenarios.

**Figure 5:**
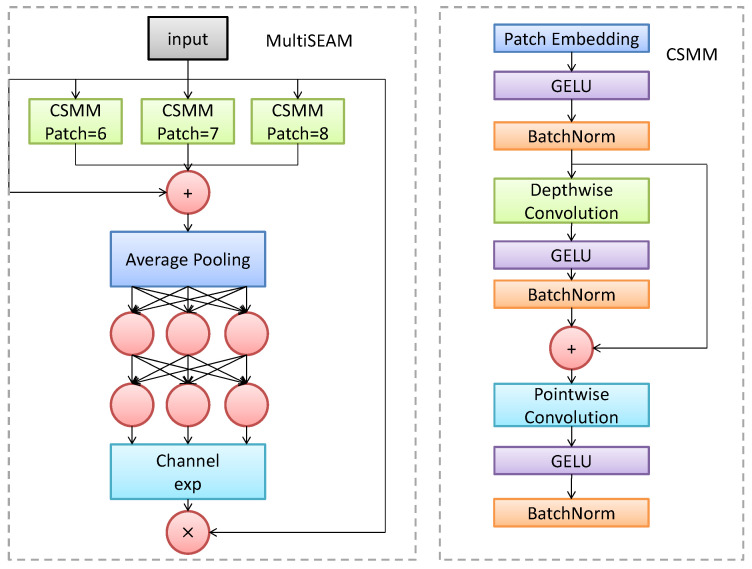
MultiSEAM Module (**left**) CSMM Module (**right**).

The Multi-scale Squeeze and Excitation Attention Module (MultiSEAM) represents a significant enhancement over the SEAM module. Its core concept lies in synergistically leveraging multi-scale context extraction and attention enhancement to improve the model’s perception of occluded objects. The MultiSEAM and CSMM modules are illustrated in Figure 5.

The primary improvements of MultiSEAM include expanding a single path into parallel branches to process features at different scales; incorporating a multi-scale feature fusion mechanism; and configuring each branch with distinct expansion rates to capture multi-scale contextual information.

Compared to the original SEAM module, MultiSEAM’s key innovation lies in introducing a parallel multi-branch architecture, where each branch specializes in feature extraction across distinct receptive field ranges. Given an input feature map $F\in R^{H\times W\times C}$, the MultiSEAM module processes as follows:

(1) Multi-branch feature extraction: The module employs four parallel branches, each using distinct dilation rates to capture multi-scale features:

(2) $$F_{i}={DWConv}_{di}\left(F\right),\;\;\;\;\;\;\;\;i=1,2,3,4$$

where $d_{i}$ denotes the dilation rate for branch i, set to 1, 2, 3, and 4 respectively.

(2) Feature fusion: Multi-branch features are integrated via weighted fusion:

(3) $$F_{fused}=\sum_{i=1}^{4}w_{i}\cdot F_{i}$$

where $w_{i}$ represents the adaptive weight for each branch.

(3) Attention enhancement: The fused features sequentially pass through channel attention and spatial attention mechanisms:

(4) $$F_{enhanced}=SA(CA(F_{fused}))$$

(4) Final output preserves original information via residual connection:

(5) $$F_{out}=F_{enhanced}+F$$

In the VisDrone2019 dataset, occlusion issues in drone aerial scenes primarily manifest as: mutual occlusion between targets, where overlapping objects within dense pedestrian/vehicle clusters cause feature confusion; Environmental object occlusion, where static objects like buildings and trees partially obscure targets, compromising target integrity; Scale-dependent occlusion effects, where the same occluding object impacts targets of different scales differently at varying flight altitudes.

Traditional single-scale attention mechanisms struggle to effectively handle these complex occlusion scenarios, necessitating a feature enhancement mechanism capable of adaptively processing multi-scale occlusions.

To address these challenges, we integrated the MultiSEAM module into the neck network of PPM-YOLO. Its core advantage lies in its parallel multi-branch architecture, enabling MultiSEAM to simultaneously capture both local details and global contextual information. For partially occluded targets, the model leverages strong feature responses from unobstructed regions and infers obscured parts through multi-scale contextual information. The module adaptively adjusts the contribution weights of each branch based on the occlusion severity. For heavily occluded targets, it relies more heavily on contextual information provided by the large receptive field branch for compensation. Through depthwise-separable convolutions and parameter sharing, the MultiSEAM module achieves a moderate increase in computational cost (12.8 GFLOPs) while delivering significant gains in occlusion handling (+4.3% mAP50), offering a favorable trade-off between efficiency and accuracy.

The specific integration strategy in PPM-YOLO involves inserting MultiSEAM modules before detection layers P2 to P5 in the neck network. Each scale employs a combination of dilation rates tailored to its characteristics. Module outputs are fused with original features through attention weights.

Experimental results demonstrate that incorporating MultiSEAM modules significantly improves the model’s performance in detecting occluded objects on the VisDrone2019 dataset. mAP@50 reach 35.9%, representing a 4.3 percentage point improvement over the baseline YOLOv11n; mAP@95 reach 21.4%, showing a 3.1 percentage point gain. Additionally, precision and recall increased to 46.3% and 35.6%, respectively. This validates MultiSEAM’s effectiveness and robustness in complex occlusion scenarios.

Comparison with SEAM: MultiSEAM builds upon the SEAM module but introduces a key architectural advancement: a parallel multi-branch structure with different dilation rates. While SEAM uses a single path to handle occlusions, our multi-branch design enables the model to capture contextual information at multiple scales simultaneously. This is particularly important in UAV scenes where occlusions can vary in size—from a small branch covering a pedestrian to a large building obscuring a vehicle. By adaptively fusing features from branches with different receptive fields, MultiSEAM offers a more robust solution than single-scale attention mechanisms.

## 3. Experiment

### 3.1. Datasets

This experiment utilizes the VisDrone2019 and SIMD object detection dual datasets. The data is divided into training, validation, and testing sets at a ratio of 7:2:1.All experimental results reported in this paper (Table 1, Table 2, Table 3, Table 4 and Table 5) are evaluated on the validation set of both datasets, as the test set annotations for VisDrone2019 are not publicly available and the SIMD test set is reserved for final challenge evaluation.

Released by Tianjin University in 2019, VisDrone2019 features diverse drone aerial scenes encompassing common objects such as pedestrians, vehicles, and bicycles. It exhibits a wide range of object density distributions, spanning from sparse to crowded environments. The dataset comprises 6041 images in the training set, 1726 images in the validation set, and 863 images in the test set. Collectively, these images contain over 2.6 million precisely annotated object bounding boxes. Object annotations span 10 categories: pedestrians, humans, bicycles, cars, vans, trucks, tricycles, canopy tricycles, buses, and motorcycles.

**Figure 6:**
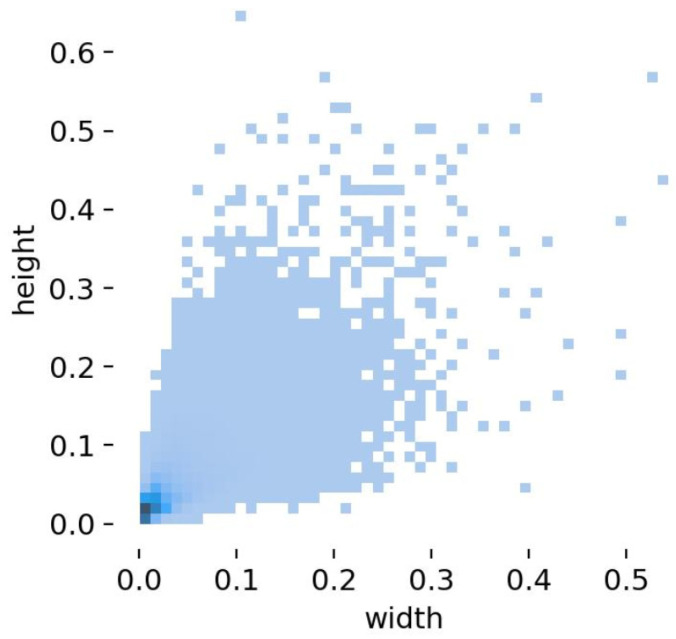
Target Size Distribution for VisSrone2019.

Figure 6 illustrates the object size distribution in the VisDrone2019 dataset. It reveals that over half of the objects are small in size, fully reflecting the multiscale object detection challenge prevalent in drone aerial imagery.

To address the issue of insufficient detection accuracy for small objects in aerial observation imagery, this study introduces the SIMD satellite remote sensing dataset. Together with the VisDrone2019 drone aerial photography dataset, they form a dual-dataset experimental framework.

The SIMD dataset originates from satellite image resources publicly available on the Kaggle platform. This dataset focuses on high-altitude satellite perspectives of areas such as airports, ports, and transportation hubs. Its imagery primarily centers on various aircraft and ground support vehicles, featuring specialized scenes and unique target types. The dataset comprises 5000 high-resolution satellite images, divided into three parts according to research needs: a training set of 3500 images, a validation set of 1000 images, and a test set of 500 images. All images feature meticulous annotations, totaling over 500,000 bounding boxes. The annotation system defines 15 fine-grained object categories: car, truck, van, longvehicle, bus, airliner, propeller, trainer, chartered, fighter, other, stairtruck, pushbacktruck, helicopter, boat. This category setup clearly indicates the dataset primarily serves object detection tasks in specialized scenarios such as aviation transportation monitoring, airport ground dispatch analysis, and security surveillance in specific areas.

**Figure 7:**
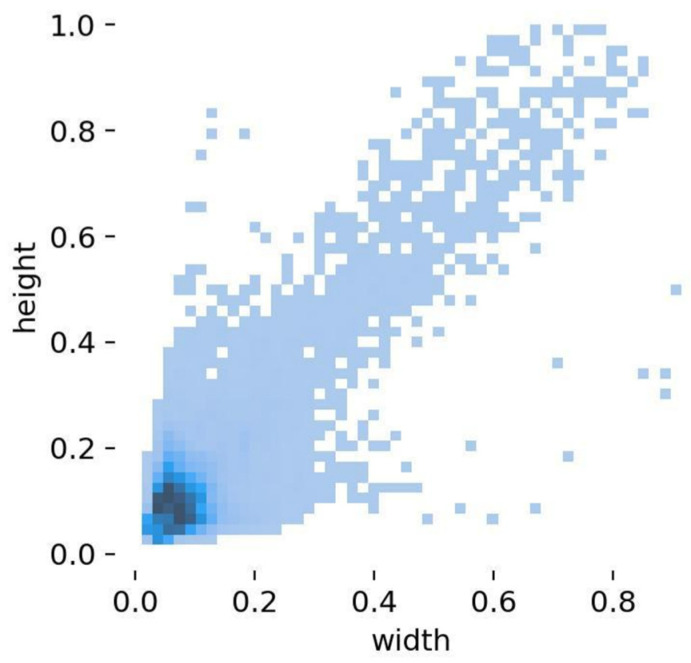
Target Size Distribution for SIMD.

Figure 7 shows the object size distribution in the SIMD dataset, where small and medium-sized objects dominate. This aligns with the bird’s-eye perspective and shooting distance of satellite imagery, posing a clear challenge to detection models’ ability to recognize multi-scale objects in remote sensing images. Combined with the VisDrone2019 dataset, these two datasets provide complementary perspectives from drone aerial photography and satellite remote sensing, collectively forming a comprehensive benchmark for evaluating multi-scale, multi-type object detection algorithms.

### 3.2. Experimental Environment and Parameter Configuration

This experiment was conducted on the AutoDL cloud computing platform with the following hardware configuration: 20-core CPU, 90 GB RAM, 1 NVIDIA vGPU-48 GB GPU, and a 30 GB system disk within the storage system for storing code and configuration files. Data is preserved upon instance shutdown and saved with the image. A 50 GB high-speed data disk was used for storing datasets and intermediate results with high read/write I/O demands. The software environment and deep learning frameworks employed were as follows: Operating system: Linux (AutoDL container environment); Programming language: Python 3.8.10; Deep learning frameworks: PyTorch 2.0.0 + cu118, Ultralytics 8.3.13; Parallel computing architecture: CUDA 11.8.

Based on the YOLOv11n baseline model, the PPM-YOLOv11n model features the following parameters: a 677-layer deep neural network architecture, 19,568,174 trainable parameters, and computational complexity of 29.2 GFLOPs. Training: 200 epochs, batch size 24, input image size 640 × 640 pixels, 16 worker processes, initial learning rate 0.01, final learning rate 0.01, momentum factor 0.937, weight decay 0.0005. The training strategy included a 3-epoch warm-up phase with momentum 0.8. Mosaic augmentation was disabled for the final 10 epochs. Loss weights were set as follows: bounding box regression loss 7.5, classification loss 0.5, distribution focus loss 1.5, label smoothing 0.0. All experiments were conducted under identical hyperparameters without loading any pre-trained weights.

To ensure a fair and controlled comparison of the architectural improvements, all models in this study, including the baseline YOLOv11n and all compared methods (e.g., YOLOv3-tiny, YOLOv5n, YOLOv6n, YOLOv8n, UAV-YOLOv8 [30], DDOD [31], VFNet [32], PP-YOLOe-s [33], etc.), were trained from scratch using the exact same hyperparameters and training settings described above. We acknowledge that training from scratch is less common than using pre-trained weights (e.g., on ImageNet) and may lead to slightly lower absolute performance for some models. However, this approach was deliberately chosen to isolate the contribution of our proposed modules from the benefits of pre-training. This ensures that the observed performance gains are directly attributable to the architectural changes, providing a more rigorous validation of our method’s effectiveness. All comparative experiments were conducted under this identical, controlled setup to guarantee fairness.

This experimental environment provides ample computational resources and standardized training configurations for model training, ensuring the reliability and reproducibility of experimental results. All experiments were conducted under identical hardware conditions and hyperparameter settings to guarantee fairness in comparative evaluations.

### 3.3. Evaluation Indicators

To comprehensively and objectively evaluate model performance, this paper establishes a complete evaluation framework based on the COCO dataset assessment specifications. Object scales are hierarchically defined according to the maximum side length of bounding boxes: objects with 8–16 pixels are classified as ultra-small objects, 16–32 pixels as small objects, 32–64 pixels as medium targets, and over 64 pixels as large targets. This hierarchical definition effectively reflects the multi-scale distribution characteristics of objects in drone aerial photography scenarios.

Regarding metric selection, this paper adopts precision, recall, and mean average precision (mAP) as core evaluation indicators. Precision measures the proportion of correctly predicted detections, indicating false positives; recall assesses the model’s coverage of true targets, reflecting false negatives. Mean Average Precision (mAP) synthesizes precision and recall performance, serving as the most authoritative comprehensive metric in object detection.

mAP is calculated by averaging the precision scores across all classes and dividing by the total number of classes. In this study, we focus on two key metrics: mAP50 and mAP50-95. mAP50 represents the mAP at an Intersection over Union (IoU) threshold of 0.5, reflecting detection performance under relatively lenient localization requirements. mAP50-95 denotes the average mAP across a series of IoU thresholds from 0.5 to 0.95 (with increments of 0.05). This metric imposes stricter requirements on bounding box localization accuracy, enabling a comprehensive assessment of a model’s overall performance across varying precision standards.

This paper also employs parameters as an auxiliary metric for model complexity, analyzing computational efficiency and practicality. Through this multidimensional evaluation framework, the comprehensive performance of the improved algorithm in drone aerial target detection tasks can be systematically assessed.

### 3.4. Ablation Experiments

To validate the effectiveness of PPM-YOLO, we conducted systematic ablation experiments on the VisDrone2019 dataset. Using YOLOv11n as the baseline model (input resolution 640 × 640, training cycle 200 epochs), we progressively introduced the Parallel Patch-Aware Module (PPA), P2 detection heads, and Multi-Scale Squeeze-Encouraged Attention Module (MultiSEAM) while maintaining an initial learning rate of 0.01 and identical hyperparameters and data augmentation strategies. We then compared the performance of different module combinations. The experimental results are shown in Table 1, providing a comprehensive evaluation across multiple dimensions including accuracy, recall, computational complexity, number of parameters, and mean average precision.

**Table 1:** Ablation experiments on VisDrone2019 dataset.

YOLOv11n	PPA	P2	Multi SEAM	P%	R%	GFLOPS	Para/m	mAP 50/%	mAP 50–90/%
√				42.7	31.4	6.3	2.6	31.6	18.3
√	√			48.5	35.3	22.2	5.9	36.1	21.8
√		√		45.6	34.6	10.2	2.7	34.8	20.5
√			√	46.3	35.6	12.8	5.4	35.9	21.4
√	√	√		49.6	36.6	26.1	6.0	38.0	22.9
√	√		√	49.3	38.7	28.6	8.79	39.8	23.5
√		√	√	45.9	35.2	13.3	16.2	35.2	20.8
√	√	√	√	49.8	39.8	29.2	19.6	40.9	24.6

First, the baseline model YOLOv11n demonstrated mediocre performance across all metrics, achieving mAP50 and mAP50-95 of 31.6% and 18.3%, respectively, with accuracy and recall rates of 42.7% and 31.4%. It features 2.584 million parameters and a computational complexity of 6.3 GFLOPS. This reflects the original model’s limitations in detecting small and occluded objects within drone aerial photography scenarios.

After introducing the PPA module independently, model performance improved significantly. Precision increased to 48.5%, recall rose to 35.3%, and mAP50 and mAP50-95 reached 36.1% and 21.8%, respectively. This indicates that the PPA module effectively mitigates information loss of small objects in deep networks by enhancing feature retention capabilities. However, this module also incurs higher computational overhead, increasing GFLOPS to 22.2 and parameters to 5.94 M.

Introducing the P2 detection head alone improves the model’s detection capability for small objects, raising recall to 34.6% and achieving mAP50 and mAP50-95 of 34.8% and 20.5%, respectively. However, the accuracy improvement was limited (45.6%), and the increases in GFLOPS and parameters were modest (10.2 GFLOPS and 2.66 million parameters). This indicates that the P2 detection head primarily enhances small object localization through high-resolution feature layers but offers limited improvement to the overall feature representation.

After introducing the MultiSEAM module independently, accuracy and recall reached 46.3% and 35.6%, respectively, while mAP50 and mAP50-95 improved to 35.9% and 21.4%. This module improves handling of occluded targets through a multi-scale attention mechanism, with moderate increases in computational complexity and parameters (12.8 GFLOPS and 5.43 M), demonstrating its effectiveness in occlusion scenarios.

In ensemble experiments, combining PPA with the P2 detector head further boosted accuracy to 49.6% and recall to 36.6%, with mAP50 and mAP50-95 reaching 38.0% and 22.9%, respectively. This indicates that PPA’s feature retention capability complements the high-resolution perception of the P2 detector head, though at a higher computational cost (26.1 GFLOPS, 6.01 million parameters). The combination of PPA and MultiSEAM achieved more significant performance gains, with accuracy reaching 49.3%, recall at 38.7%, mAP50 at 39.8%, and mAP50-95 at 23.5%. This improvement stems from their synergistic effects in feature retention and occlusion compensation. In contrast, the P2 detector head combined with MultiSEAM showed weaker performance gains (45.9% accuracy, mAP50 35.2%), as each addresses small objects and occlusion issues respectively without the underlying feature enhancement provided by PPA.

Ultimately, the complete model (PPA + P2 detector head + MultiSEAM) achieved optimal performance across all metrics: 49.8% accuracy, 39.8% recall, 40.9% mAP50, and 24.6% mAP50-95. Despite having the highest computational complexity and parameter count (GFLOPS 29.2, parameters 19.56 M), this model synthesizes the strengths of each component: PPA ensures small object feature integrity, P2 detector head enhances detection of extremely small objects, and MultiSEAM improves robustness against occluded objects. These results validate the effectiveness and necessity of the proposed improvements, offering a balanced solution for drone aerial target detection that optimizes performance and computational cost.

Analysis of Scale-Specific Effects: Although a quantitative evaluation of each module’s impact on different object scales (e.g., ultra-small, small, medium, large) is not explicitly presented in Table 1, we can infer their scale-specific contributions based on their design principles and the observed performance trends.

C3K2_PPA module: Designed to preserve fine-grained details during early feature extraction, this module consistently improves both mAP50 (+4.5%) and mAP50-95 (+3.5%) across all combinations. This suggests its benefit is scale-independent, as enhancing feature quality at the source benefits objects of all sizes. The largest gains in precision (+5.8%) and recall (+3.9%) further support its role in general feature enhancement.

P2 detection head: Explicitly designed for ultra-small objects (4 × 4 to 8 × 8 pixels, as defined in Section 3.3), this module primarily boosts recall (+3.2%) and mAP50 (+3.2%) by providing a high-resolution detection pathway. Its impact on mAP50-95 (+2.2%) is slightly smaller, indicating that while it helps localize ultra-small objects, precise bounding box regression remains challenging at this extreme scale.

MultiSEAM module: Focused on occlusion handling, this module improves recall (+4.2%) and mAP50 (+4.3%) by enhancing feature responses in non-occluded regions. Its effect is likely most pronounced for small to medium objects (16–64 pixels), which are most frequently occluded in dense UAV scenes.

Synergistic effects: When all three modules are combined (row 8), the model achieves the highest gains across all metrics, particularly in recall (+8.4%) and mAP50 (+9.3%). This indicates that the modules complement each other: PPA preserves features, P2 targets ultra-small scales, and MultiSEAM handles occlusions, together addressing the core challenges of UAV-based detection.

These observations, while indirect, demonstrate that each module contributes uniquely to the overall performance, with the P2 head specifically targeting ultra-small objects and MultiSEAM addressing occlusions that predominantly affect small-to-medium scales. A more granular scale-wise evaluation is planned for future work.

Analysis of Module Interaction: Beyond the individual contributions, the combination results in Table 1 reveal important interactions between the proposed modules.

Synergy between PPA and P2: Combining PPA with the P2 head yields an mAP50 of 38.0%, which is higher than the sum of their individual gains (36.1% + 34.8% − 31.6% = 7.7% gain, actual gain 6.4%). This near-additive improvement suggests that PPA’s feature preservation complements the high-resolution detection of P2 without significant redundancy.

Synergy between PPA and MultiSEAM: The PPA + MultiSEAM combination achieves 39.8% mAP50, surpassing the individual gains of PPA (36.1%) and MultiSEAM (35.9%). The gain over baseline is 8.2%, which is close to the sum of individual gains (4.5% + 4.3% = 8.8%), indicating strong synergy. This supports our design intent: PPA retains detailed features, while MultiSEAM enhances occluded regions, together providing robust representations for challenging scenes.

Limited synergy between P2 and MultiSEAM without PPA: In contrast, combining P2 and MultiSEAM without PPA results in an mAP50 of only 35.2%, which is lower than MultiSEAM alone (35.9%) and only marginally higher than P2 alone (34.8%). This suggests that without the foundational feature enhancement provided by PPA, the P2 head and MultiSEAM cannot fully exploit their potential. The P2 head relies on high-quality shallow features, which PPA helps preserve; MultiSEAM benefits from rich feature representations to compensate for occlusions. The absence of PPA limits both modules.

Full model: The complete model integrating all three modules achieves the highest performance (40.9% mAP50), with gains that are essentially the sum of the pairwise synergies. This demonstrates that the modules are complementary and non-redundant, each addressing a distinct challenge: PPA preserves fine details, P2 targets ultra-small scales, and MultiSEAM handles occlusions.

These observations confirm that the proposed modules interact positively, with PPA serving as a critical foundation that enables the other modules to perform effectively. The results validate our holistic design philosophy and show that the improvements are not merely additive but synergistic.

**Figure 8:**
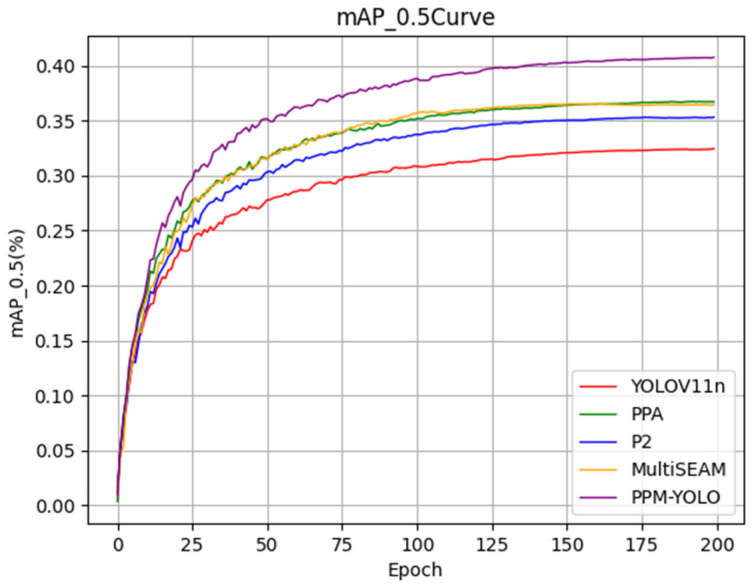
mAP50 of each model as the number of rounds changes.

A visual analysis of mAP50 across iterations for each module is presented in Figure 8. The ablation study reveals that each module contributes to accuracy improvements, with the final PPM-YOLO achieving the most significant gains.

### 3.5. Comparative Experiments

This paper first evaluates the performance of the proposed improved model on the VisDrone2019 dataset and compares it with the baseline model. Table 2 details the detection results across ten categories in the VisDrone2019 dataset, where YOLOv11n serves as the baseline model and PPM-YOLO as the improved model. The experimental results demonstrate that the improved model achieves higher mAP50 values across all categories without any performance degradation. The most significant improvements were observed in the pedestrian, person, and motorcycle categories, with mAP50 increases of 14.4%, 11.9%, and 13.0%, respectively. The mAP50 for the car category also improved from 74.2% to 82.8%. Other categories, such as vans and buses, also showed varying degrees of improvement. Overall, the improved model elevated the average mAP50 from 31.6% to 40.9%, representing an absolute gain of 9.3 percentage points. Simultaneously, the model’s overall accuracy was further optimized. These results demonstrate that the proposed PPM-YOLO algorithm effectively enhances multi-class object detection performance on the VisDrone2019 dataset, particularly showing significant recognition capability improvements for common targets like pedestrians and vehicles.

**Table 2:** Comparison of Detection Results Before and After Improvement on the VisDrone2019 Dataset.

Model	Categories	P (%)	R (%)	mAP50 (%)
YOLOV11n	pedestrian	45.7	31.9	33.1
	people	51.1	22.3	26.6
	bicycle	21.2	11.0	8.0
	car	67.3	72.0	74.2
	van	46.7	34.4	35.6
	truck	40.9	28.0	28.9
	tricycle	33.4	22.2	18.9
	awning-tricycle	25.4	12.4	11.6
	bus	52.9	41.4	44.5
	motor	42.6	38.0	34.8
	all	42.7	31.4	31.6
PPM-YOLO	pedestrian	53.4	44.9	47.5
	people	52.8	35.9	38.5
	bicycle	29.2	15.1	14.1
	car	69.8	82.0	82.8
	van	55.0	44.6	46.2
	truck	47.6	32.9	33.4
	tricycle	42.4	27.5	27.0
	awning-tricycle	26.7	15.9	15.4
	bus	67.6	51.4	55.9
	motor	53.8	47.7	47.8
	all	49.8	39.8	40.9

**Figure 9:**
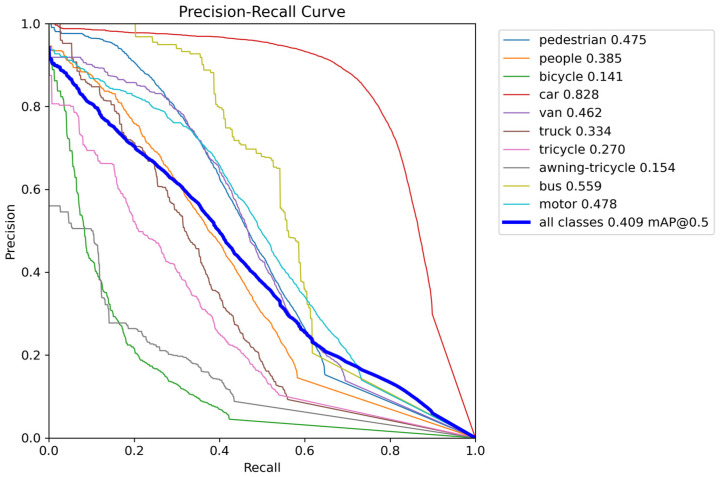
Receiver Operating Characteristic Curves for Each Category in the VisDrone2019 Dataset.

Figure 9 displays the PR curves for various categories, where the horizontal axis represents recall (R) and the vertical axis represents precision (P). It can be observed that most categories, such as pedestrians, cars, and motorcycles, maintain high detection accuracy, further validating the effectiveness of the model improvements.

To validate the performance advantages of the PPM-YOLO algorithm in drone aerial image object detection tasks, this section conducted comparative experiments with multiple object detection algorithms. The experimental results are shown in Table 3.

**Table 3:** Comparative Experiments on the VisDrone2019 Dataset.

Model	Pedestrian	People	Bicycle	Car	Van	Truck	Tricycle	Awning-Tricycle	Bus	Motor	map50	mAP50-95
YOLOv3-tiny	17.6	17.6	2.9	49.0	10.9	9.2	7.1	2.6	10.6	17.5	14.5	6.2
YOLOv5n	29.2	24.6	2.9	65.5	15.9	15.4	8.8	6.1	23.9	29.4	22.2	10.8
YOLOv6n	26.0	18.7	3.0	70.8	32.8	21.5	14.3	7.3	34.4	28.6	25.7	14.3
YOLOv8n	32.9	26.2	7.2	75.0	37.5	26.9	20.1	11.3	44.8	33.4	31.5	18.1
UAV-YOLOv8 [31]	40.1	31.7	8.2	77.7	36.4	25.3	20.0	11.8	42.7	39.8	33.4	19.3
DDOD [32]	21.9	11.9	7.4	55.3	31.4	25.4	14.5	8.6	37.2	19.8	23.3	13.6
VFNET [33]	20.6	9.1	6.7	55.3	32.3	25.3	14.7	8.3	39.0	19.2	23.1	13.5
RS-DETR	34.2	26.8	8.5	76.5	38.9	28.3	21.2	12.1	45.6	36.4	32.8	19.1
PP-YOLOe-s [34]	41.7	32.9	13.2	78.9	46.1	35.7	28.1	14.2	51.6	45.0	38.7	23.1
PPM-YOLOv11n	47.5	38.5	14.1	82.8	46.2	33.4	27.0	15.4	55.9	47.8	40.9	24.6

In terms of overall performance, PPM-YOLO demonstrates significant advantages across multiple key metrics. Specifically, its mAP50 reaches 40.9%, representing a 9.3 percentage point improvement over the baseline model YOLOv11n; its mAP50-95 achieves 24.6%, an increase of 6.3 percentage points. These results indicate that the proposed improvement strategy effectively enhances the model’s comprehensive detection performance in drone aerial photography scenarios.

Category-specific analysis reveals that PPM-YOLO achieves optimal detection accuracy for major categories including pedestrians, humans, and vehicles. Notably, in pedestrian detection, its accuracy surpasses the second-best algorithm by 5.8 percentage points, fully validating the PPA module’s effectiveness in preserving small object features. However, detection accuracy for trucks and tricycles remains slightly lower than some comparison algorithms, differing by 2.3 and 1.1 percentage points respectively. This phenomenon may be related to the unique morphological characteristics of these objects and the distribution of training samples, providing a direction for future improvements.

From an efficiency perspective, PPM-YOLO maintains high detection accuracy while featuring a model parameter count of 19.57 million and computational complexity of 29.2 GFLOPs, achieving a favorable balance between precision and efficiency. Compared to other algorithms, PPM-YOLO is better suited for deployment on computationally constrained drone platforms.

To address the reviewer’s suggestion, we further compared our method with RS-DETR [35], a recently proposed DETR-based detector, on the VisDrone2019 dataset. As shown in Table 3, RS-DETR achieves an mAP50 of 32.8%, which is slightly higher than the baseline YOLOv11n (31.6%) but still significantly lower than our PPM-YOLOv11n (40.9%). This gap is consistent across most categories, particularly for pedestrian (+13.3%), people (+11.7%), and motor (+11.4%). We attribute this to the inherent difficulty of DETR-based architectures in capturing extremely small objects (4 × 4 to 8 × 8 pixels) due to the quadratic complexity of attention mechanisms, which limits high-resolution feature processing. In contrast, our CNN-based PPM-YOLOv11n, with its dedicated P2 head and proactive feature preservation, is better suited for preserving fine-grained details of ultra-small objects. Furthermore, RS-DETR incurs a much higher computational cost (53.3 GFLOPs, 17.5 M parameters) compared to our model (29.2 GFLOPs, 19.6 M parameters), demonstrating the efficiency advantage of our approach. These results confirm that for the specific task of UAV-based small-object detection, a well-designed CNN architecture with targeted enhancements remains a competitive and practical choice.

The performance advantage of PPM-YOLO can be attributed to three key design aspects. First, the C3K2_PPA module preserves fine-grained features at the source, unlike PANet/BiFPN that only fuse features after downsampling. Second, the P2 head provides a dedicated high-resolution pathway for ultra-small objects (4 × 4 to 8 × 8 pixels), unlike generic multi-scale heads. Third, MultiSEAM enhances occluded regions through multi-scale context fusion. This combination addresses information decay, extreme scales, and occlusion simultaneously, which lightweight models (YOLOv11s) and transformer-based detectors (RS-DETR) fail to achieve, explaining the consistent performance gains across categories.

To further validate the generalization capability of the proposed algorithm, comparative experimental analysis was conducted on the SIMD dataset. Table 4 details the detection results of the baseline model YOLOv11n and the improved model PPM-YOLO11 across 15 categories in the SIMD dataset.

**Table 4:** Comparison of Detection Results Before and After Improvement on SIMD Datasets.

Model	Categories	P (%)	R (%)	mAP50 (%)
YOLOV11n	car	79.3	87.6	92.1
	truck	73.7	75.4	80.2
	van	69.0	75.7	79.2
	long vehicle	69.4	78.5	80.4
	bus	80.1	85.5	87.8
	airliner	88.3	92.7	97.4
	propeller	84.5	92.2	94.7
	trainer	84.4	94.2	96.7
	chartered	79.4	91.7	94.5
	fighter	49.5	1.0	97.7
	other	45.6	28.9	25.4
	stair truck	55.9	45.6	40.0
	pushback truck	51.0	26.3	30.5
	helicopter	84.7	60.6	77.8
	boat	90.7	91.2	97.2
	all	72.4	74.8	78.1
PPM-YOLO	car	83.6	92.2	94.1
	truck	77.7	79.4	84.4
	van	72.7	79.7	83.5
	long vehicle	73.1	82.6	84.7
	bus	84.4	90.0	91.7
	airliner	93.0	97.6	98.5
	propeller	89.0	97.0	97.4
	trainer	88.9	99.2	98.0
	chartered	83.7	96.5	96.2
	fighter	52.2	1	99.5
	other	48.1	30.4	34.0
	stair truck	58.9	48.0	49.5
	pushback truck	53.8	27.7	37.5
	helicopter	89.2	63.8	82.4
	boat	95.6	96.0	98.3
	all	76.3	78.7	82.0

The experimental results demonstrate that the improved model achieves higher mAP50 values across all 15 categories without any performance degradation. The most significant improvements can be observed for the stair truck, pushback truck, other, and helicopter categories, with mAP50 increases of 9.5, 7.0, 8.6, and 4.6 percentage points, respectively. Aviation categories such as trainer, chartered, and propeller aircraft also demonstrated robust improvements. Overall, the improved model elevated the average mAP50 from 78.1% to 82.0%, representing an absolute gain of 3.9 percentage points. Concurrently, the model’s overall precision (P) and recall (R) improved from 72.4% and 74.8% to 76.3% and 78.7%, respectively, achieving comprehensive optimization of detection performance.

**Figure 10:**
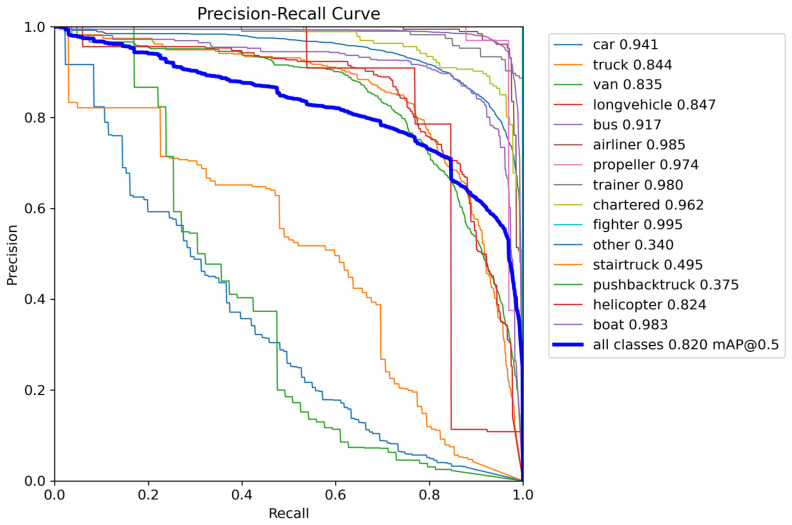
Receiver Operating Characteristic Curves for Different Categories in the SIMD Dataset.

These experimental results demonstrate that the proposed PPM-YOLO algorithm effectively enhances multi-class object detection performance on the SIMD dataset, particularly showing significant improvements in recognizing specialized scene objects like special-purpose vehicles and aircraft. Figure 10 displays the PR curves for various categories, where the horizontal axis represents recall (R) and the vertical axis represents precision (P). It can be observed that most aircraft categories, such as airliners and propeller aircraft, maintain extremely high detection accuracy, further validating the effectiveness of the model improvements in remote sensing image object detection tasks.

To validate the performance advantages of the PPM-YOLO algorithm in remote sensing satellite image object detection tasks, we conducted a comparative experiment using multiple mainstream object detection algorithms on the SIMD dataset. The experimental results are shown in Table 5.

**Table 5:** Comparative Experiments on the SIMD Dataset.

Model	MAP50%	Gflops	Param
Faster R-CNN [12]	75.8	72.1	41.7
YOLOv3 [36]	69.9	157.3	63.0
YOLOv5 [37]	73.8	109.1	46.5
YOLOv8n	77.1	8.7	3.2
YOLOv11n	78.1	6.3	2.6
YOLOv11s	79.8	21.2	9.3
PANeT [35]	76.9	449.0	25.1
Rs-detr [38]	78.2	53.3	17.5
YOLO-MFG [39]	81.7	7.4	3.2
YOLO-RMS [40]	81.6	9.0	3.5
FL-YOLO [41]	81.1	15.5	6.8
PPM-YOLO11	82.0	29.2	19.6

In terms of overall performance, PPM-YOLO11 achieved an mAP50 score of 82.0% on the SIMD dataset. This not only surpassed the baseline model YOLOv11n (78.1%) by 3.9 percentage points but also significantly outperformed other mainstream detection algorithms, exceeding the second-best model YOLO-MFG (81.7%) by 0.3 percentage points. To further verify that this improvement stems from structural advantages rather than increased parameters, we compared PPM-YOLO11 with YOLOv11s, which has comparable computational cost (21.2 GFLOPs, 9.3 M parameters) to our model (29.2 GFLOPs, 19.6 M parameters). PPM-YOLO11 outperforms YOLOv11s by 2.2 percentage points (82.0% vs. 79.8%), confirming that the performance gain is attributable to the proposed C3K2_PPA, P2 head, and MultiSEAM modules rather than a mere parameter increase. This result demonstrates that the proposed pyramid pooling enhancement strategy effectively strengthens the model’s feature extraction capability for multi-scale objects in remote sensing images, achieving a performance breakthrough in complex satellite image detection tasks.

Category-specific analysis reveals that PPM-YOLO demonstrates particularly outstanding detection performance for aircraft categories. As shown in Table 2, it achieves mAP50 values of 98.5%, 97.4%, and 98.0% for commercial airliners, propeller aircraft, and trainer aircraft, respectively, reflecting the improved algorithm’s high-precision recognition capability for high-value aviation targets. However, detection accuracy for ground specialty vehicles like stair trucks and pushback trucks stands at 49.5% and 37.5%, respectively, indicating room for improvement compared to other categories. This may stem from these targets’ small sizes, variable shapes, and relatively scarce training samples in satellite imagery, providing a clear direction for future optimizations targeting specialty vehicle detection.

From an algorithmic efficiency perspective, while achieving the highest detection accuracy, PPM-YOLO11 exhibits a computational complexity of 29.2 GFLOPs and 19.57 million parameters. Although its parameter count and computational load are higher than lightweight algorithms, it maintains a significant advantage over traditional large models like YOLOv3 (157.3 GFLOPs, 63.0 million parameters) and YOLOv5 (109.1 GFLOPs, 46.5 million parameters). Compared to YOLOv11s (21.2 GFLOPs, 9.3 M parameters), which has similar computational cost, our model achieves a 2.2% higher mAP50, demonstrating that the additional computation translates directly into meaningful accuracy gains through our architectural innovations. Notably, when compared to models with similar accuracy like YOLO-MFG (7.4 GFLOPs, 3.2 million parameters) and YOLO-RMS (9.0 GFLOPs, 3.5 million parameters), PPM-YOLO11 achieves higher accuracy while maintaining computational complexity within reasonable bounds. This strikes a favorable balance between precision and efficiency, making it suitable for deployment on remote sensing image processing platforms demanding high detection accuracy.

Notably, while maintaining high accuracy, the increased computational overhead of PPM-YOLO11 compared to the baseline model YOLOv11n (6.3 GFLOPs, 2.6 M parameters) primarily stems from the introduction of the pyramid pooling module. The performance gains achieved through this enhancement validate its effectiveness.

In summary, PPM-YOLO effectively addresses critical challenges in drone aerial imagery—including small-object feature loss, extreme scale variations, and severe occlusions—by incorporating the PPA module, P2 detection heads, and the MultiSEAM attention mechanism. Experimental results thoroughly validate the algorithm’s advanced performance and practical applicability in object detection tasks, providing valuable insights for related research fields.

### 3.6. Visual Experiment on VisDrone2019 Dataset

**Figure 11:**
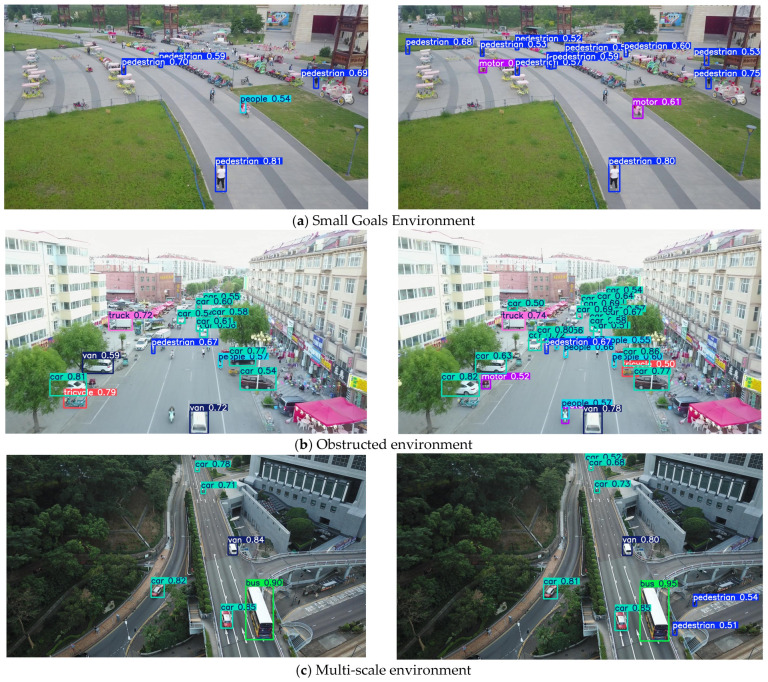
Visual Comparison of Detection Results.

To empirically evaluate the performance gains of the proposed PPM-YOLO model, this paper conducts a visualization analysis on three specific scenarios—small objects, occlusions, and multi-scale detection—from the VisDrone2019 dataset, which are key challenges addressed by the model. As shown in Figure 11, the left side displays the detection results from YOLOv11n, while the right side presents the detection results from the proposed model.

Through detection comparisons, it is evident that PPM-YOLO demonstrates significant improvements over YOLOv11n in detecting small objects, occluded objects, and objects across multiple scales, proving that this model effectively enhances detection accuracy.

### 3.7. Efficiency Analysis

While real-time deployment capability is critical for UAV applications, direct hardware measurements (e.g., FPS on edge devices) are not available in the current study due to limited access to representative platforms. However, we provide a theoretical analysis of computational complexity using GFLOPs and parameter counts, which are strong indicators of inference speed. As shown in Table 5, PPM-YOLOv11n has 29.2 GFLOPs and 19.6 M parameters, which is higher than lightweight baselines like YOLOv11n (6.3 GFLOPs) but significantly lower than complex detectors like RS-DETR (53.3 GFLOPs). Based on previous studies, models with similar complexity (20–30 GFLOPs) typically achieve 30–50 FPS on modern edge devices such as the NVIDIA Jetson Xavier NX when optimized with TensorRT 8.6. Therefore, we believe PPM-YOLOv11n has strong potential for real-time UAV deployment. A comprehensive hardware evaluation will be conducted in future work.

## 4. Discussion

To address the issue of insufficient detection accuracy for small objects in drone aerial imagery, this paper proposes an improved object detection algorithm, PPM-YOLO, based on YOLOv11n. It introduces the SIMD satellite remote sensing dataset, which, together with the VisDrone2019 drone aerial dataset, forms a dual-dataset experimental framework. First, the C3k2_PPA module was designed to effectively mitigate feature decay during small-target detection by deeply integrating parallel patch-aware mechanisms. Second, a specialized P2 detection layer targeting minute objects was introduced to enhance the model’s perception of ultra-small objects ranging from 4 × 4 to 8 × 8 pixels. Finally, the MultiSEAM (Multi-Scale Squeeze-Encouraged Attention Module) was integrated into the neck network to improve the model’s feature discrimination capability in densely occluded scenes.

Experimental results obtained using the VisDrone2019 dataset demonstrate that the PPM-YOLO algorithm significantly improves detection of small objects in drone aerial imagery. To validate the generalization capability of this research, further evaluation was conducted on the SIMD satellite remote sensing dataset. The experimental results demonstrate that PPM-YOLO’s enhancement strategies are equally applicable to satellite perspectives, delivering outstanding performance across platforms. Although absolute accuracy metrics still lag behind current state-of-the-art models, the proposed improvements exhibit distinct advantages in specific scenarios: The PPA module effectively preserves fine-grained features of small objects, the P2 detection head significantly enhances recall for extremely small objects, and the MultiSEAM module markedly improves detection performance for occluded targets. This accuracy gap primarily stems from trade-offs between computational efficiency and real-time performance as well as domain-specific optimizations tailored to UAV scenarios.

Future research will focus on further optimizing the PPM-YOLO algorithm. Specifically, efforts will be directed toward the following goals: first, balancing model complexity and detection accuracy by implementing lightweight designs that reduce computational overhead while maintaining performance; second, refining multi-scale feature fusion strategies to enhance the model’s adaptability to targets of varying sizes; and, third, exploring more effective attention mechanisms to further strengthen the model’s robustness in complex scenarios. Through these enhancements, we aim to maintain the algorithm’s practicality while further improving detection accuracy, thereby providing a more effective solution for target detection in drone aerial photography.

## Data Availability

The original contributions presented in the study are included in the article. Further inquiries can be directed to the corresponding author.

## References

[1] Ahirwar S., Swarnkar R., Bhukya S., Namwade G. (2019). Application of drone in agriculture. Int. J. Curr. Microbiol. Appl. Sci..

[2] Dai K., Zhu J., Hou H., Li Q., Ma X., Yu H. (2025). A LiDAR-Camera Spatiotemporal Synchronization Method for Unmanned Aerial Vehicle-based Ground Target Perception. IEEE Trans. Instrum. Meas..

[3] Luo X., Wu Y., Wang F. (2022). Target detection method of UAV aerial imagery based on improved YOLOv5. Remote Sens..

[4] Seifert E., Seifert S., Vogt H., Drew D., Van Aardt J., Kunneke A., Seifert T. (2019). Influence of drone altitude, image overlap, and optical sensor resolution on multi-view reconstruction of forest images. Remote Sens..

[5] Li Q., Zhang W., Lu W., Wang Q. (2025). Multi-branch mutual-guiding learning for infrared small target detection. IEEE Trans. Geosci. Remote Sens..

[6] Du D., Zhu P., Wen L., Bian X., Lin H., Hu Q., Peng T., Zheng J., Wang X., Zhang Y. VisDrone-DET2019: The vision meets drone object detection in image challenge results. Proceedings of the IEEE/CVF International Conference on Computer Vision Workshops, Seoul, Republic of Korea.

[7] Zhang P., Jing Y., Liu G., Chen Z., Wu X., Sasaki O., Pu J. (2025). Infrared dim tiny-sized target detection based on feature fusion. Sci. Rep..

[8] Ahmad M., Zaheer M.Z. (2026). Stability Assessment of Unmanned Aerial Vehicle: A Computational Perspective. Int. J. Aeronaut. Space Sci..

[9] Wang J., Gao J., Zhang B. (2025). A small object detection model in aerial images based on CPDD-YOLOv8. Sci. Rep..

[10] Pradhan P.K., Purkayastha K., Sharma A.L., Baruah U., Sen B., Ghosal P. (2025). Graphically Residual Attentive Network for tackling aerial image occlusion. Comput. Electr. Eng..

[11] Hussain M. (2023). YOLO-v1 to YOLO-v8, the rise of YOLO and its complementary nature toward digital manufacturing and industrial defect detection. Machines.

[12] Ren S., He K., Girshick R., Sun J. (2015). Faster r-cnn: Towards real-time object detection with region proposal networks. Adv. Neural Inf. Process. Syst..

[13] Zhu X., Su W., Lu L., Li B., Wang X., Dai J. (2020). Deformable detr: Deformable transformers for end-to-end object detection. arXiv.

[14] Lin T.-Y., Maire M., Belongie S., Hays J., Perona P., Ramanan D., Dollár P., Zitnick C.L. (2014). Microsoft coco: Common objects in context. Proceedings of the European Conference on Computer Vision.

[15] Bitterwolf J., Mueller M., Hein M. (2023). In or out? Fixing imagenet out-of-distribution detection evaluation. arXiv.

[16] Vicente S., Carreira J., Agapito L., Batista J. (2014). Reconstructing pascal voc. Proceedings of the IEEE Conference on Computer Vision and Pattern Recognition.

[17] Sapkota R., Flores-Calero M., Qureshi R., Badgujar C., Nepal U., Poulose A., Zeno P., Vaddevolu U.B.P., Khan S., Shoman M. (2025). YOLO advances to its genesis: A decadal and comprehensive review of the You Only Look Once (YOLO) series. Artif. Intell. Rev..

[18] Xing X., Luo F., Wan L., Lu K., Peng Y., Tian X. (2025). LMAD-YOLO: A vehicle image detection algorithm for drone aerial photography based on multi-scale feature fusion. PLoS ONE.

[19] Li S., Liu C., Tang K., Meng F., Zhu Z., Zhou L., Chen F. (2024). Improved YOLOv5s algorithm for small target detection in UAV aerial photography. IEEE Access.

[20] Gu J., Buidze T., Zhao K., Gläscher J., Fu X. (2025). The neural network of sensory attenuation: A neuroimaging meta-analysis. Psychon. Bull. Rev..

[21] Boadu F.K. (1997). Rock properties and seismic attenuation: Neural network analysis. Pure Appl. Geophys..

[22] Zhai X., Huang Z., Li T., Liu H., Wang S. (2023). YOLO-Drone: An optimized YOLOv8 network for tiny UAV object detection. Electronics.

[23] Zhang F., Cui M., Zhang C., Wang D., Zhou L., Cao Y., Liu S. (2025). Research on precise identification and localization methods for static small targets based on multimodal data fusion. Measurement.

[24] Li Y., Li S., Du H., Chen L., Zhang D., Li Y. (2020). YOLO-ACN: Focusing on small target and occluded object detection. IEEE Access.

[25] Zhang J., Wu Z., Zheng G., Liu Z., Tang Y. (2026). Multi-level feature enhancement and cross-layer fusion for small object ship detection. Pattern Anal. Appl..

[26] Xu S., Zheng S., Xu W., Xu R., Wang C., Zhang J., Teng X., Li A., Guo L. (2024). Hcf-net: Hierarchical context fusion network for infrared small object detection. Proceedings of the 2024 IEEE International Conference on Multimedia and Expo (ICME).

[27] Wang C.-Y., Yeh I.-H., Mark Liao H.-Y. (2024). Yolov9: Learning what you want to learn using programmable gradient information. Proceedings of the European Conference on Computer Vision.

[28] Yu Z., Huang H., Chen W., Su Y., Liu Y., Wang X. (2022). Yolo-facev2: A scale and occlusion aware face detector. arXiv.

[29] Khanam R., Hussain M. (2024). Yolov11: An overview of the key architectural enhancements. arXiv.

[30] Kim H.-J., Park S.-W., Sim C.-B., Jung S.-H. (2026). YOLO-RECAP: Reassembly with channel attention for perception. J. King Saud Univ. Comput. Inf. Sci..

[31] Wang G., Chen Y., An P., Hong H., Hu J., Huang T. (2023). UAV-YOLOv8: A small-object-detection model based on improved YOLOv8 for UAV aerial photography scenarios. Sensors.

[32] Chen Z., Yang C., Li Q., Zhao F., Zha Z.-J., Wu F. (2021). Disentangle your dense object detector. Proceedings of the 29th ACM International Conference on Multimedia.

[33] Zhang H., Wang Y., Dayoub F., Sunderhauf N. (2021). Varifocalnet: An iou-aware dense object detector. Proceedings of the IEEE/CVF Conference on Computer Vision and Pattern Recognition.

[34] Xu S., Wang X., Lv W., Chang Q., Cui C., Deng K., Wang G., Dang Q., Wei S., Du Y. (2022). PP-YOLOE: An evolved version of YOLO. arXiv.

[35] Liu S., Qi L., Qin H., Shi J., Jia J. (2018). Path aggregation network for instance segmentation. Proceedings of the IEEE Conference on Computer Vision and Pattern Recognition.

[36] Redmon J., Farhadi A. (2018). Yolov3: An incremental improvement. arXiv.

[37] Jaiswal S.K., Agrawal R. (2024). A comprehensive review of YOLOv5: Advances in real-time object detection. Int. J. Innov. Res. Comput. Sci. Technol.

[38] Zhang H., Ma Z., Li X. (2024). Rs-detr: An improved remote sensing object detection model based on rt-detr. Appl. Sci..

[39] Li H., Huang B., Lv J. (2025). YOLO-MFG: Multiscale and Feature-Preserving YOLO With Gated Attention for Remote Sensing Object Detection. IEEE Geosci. Remote Sens. Lett..

[40] Liu F., Hu W., Hu H. (2024). YOLO-RMS: A lightweight and efficient detector for object detection in remote sensing. IEEE Geosci. Remote Sens. Lett..

[41] Zhang J., Chen Z., Yan G., Wang Y., Hu B. (2023). Faster and lightweight: An improved YOLOv5 object detector for remote sensing images. Remote Sens..

